# *Cryptosporidium parvum* upregulates miR-942-5p expression in HCT-8 cells *via* TLR2/TLR4-NF-κB signaling

**DOI:** 10.1186/s13071-020-04312-x

**Published:** 2020-08-31

**Authors:** Guiling Zhang, Yajun Zhang, Ziwen Niu, Chenrong Wang, Fujie Xie, Juanfeng Li, Sumei Zhang, Meng Qi, Fuchun Jian, Changshen Ning, Longxian Zhang, Rongjun Wang

**Affiliations:** 1 College of Veterinary Medicine, Henan Agricultural University, Zhengzhou, 450046 P. R. China; 2 College of Animal Science, Tarim University, Alar, 843300 Xinjiang P. R. China

**Keywords:** *Cryptosporidium parvum*, HCT-8, TLRs, NF-κB, miR-942-5p

## Abstract

**Background:**

Micro (mi)RNAs are small noncoding RNA molecules that function in RNA silencing and post-transcriptional regulation of gene expression. This study investigated host miRNA activity in the innate immune response to *Cryptosporidium parvum* infection.

**Methods:**

*In vitro* infection model adopts HCT-8 human ileocecal adenocarcinoma cells infected with *C. parvum*. The expression of miR-942-5p was estimated using quantitative real-time polymerase chain reaction (qPCR). The TLRs-NF-κB signaling was confirmed by qPCR, western blotting, TLR4- and TLR2-specific short-interfering (si)RNA, and NF-κB inhibition.

**Results:**

HCT-8 cells express all known toll-like receptors (TLRs). *Cryptosporidium parvum* infection of cultured HCT-8 cells upregulated TLR2 and TLR4, and downstream TLR effectors, including NF-κB and suppressed IκBα (nuclear factor of kappa light polypeptide gene enhancer in B cells inhibitor, alpha). The expression of miR-942-5p was significantly upregulated at 4, 8, 12 and 24 h post-infection, and especially at 8 hpi. The results of TLR4- and TLR2-specific siRNA and NF-κB inhibition showed that upregulation of miR-942-5p was promoted by p65 subunit-dependent TLR2/TLR4-NF-κB pathway signaling.

**Conclusions:**

miR-942-5p of HCT-8 cells was significantly upregulated after *C. parvum* infection, especially at 8 hpi, in response to a p65-dependent TLR2/TLR4-NF-κB signaling. TLR4 appeared to play a dominant role.
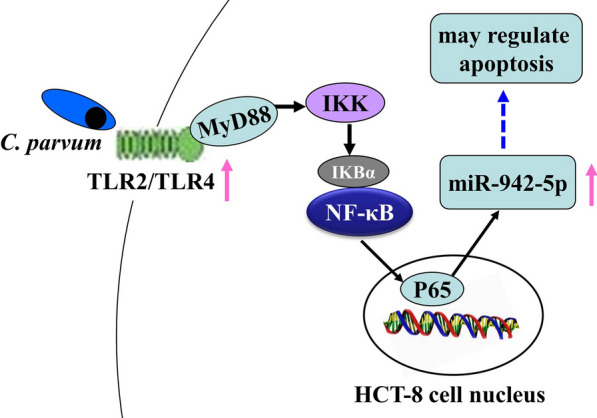

## Background

*Cryptosporidium* is an emerging zoonotic pathogen that causes diarrhea in both immunocompetent and immunosuppressed hosts, and is second only to rotavirus as a cause of moderate-to-severe diarrhea in children under two years of age [1]. In immunocompromised patients, it can cause severe, life-threatening prolonged disease. In 2016, the disease burden of cryptosporidiosis in children younger than five years was more than 12 million disability-adjusted life-years (DALYs) [2]. Thirty-eight *Cryptosporidium* valid species and approximate 60 *Cryptosporidium* genotypes have been identified or described in animals, humans, and environmental samples [3] but *C. parvum* and *C. hominis* are responsible for more than 90% of infections in humans. Despite recent efforts, effective prophylaxis and treatment are not available.

Micro (mi)RNAs are small noncoding RNA molecules found in plants, animals, viruses, and some parasite species. They function in RNA silencing and post-transcriptional regulation of gene expression [4]. Because *C. parvum* lacks key components needed for small RNA-mediated post-transcriptional gene silencing, it is an ideal model for investigating miRNA-mediated defenses against infection in epithelial cells [5]. At least seven host miRNAs, let-7i, miR-98, miR-513, miR-424, miR-503, miR-221 and miR-27b, are thought to be active in the innate immune defense against *Cryptosporidium* infection [6–13]. In biliary epithelial cells for example, *C. parvum* reduces the expression of let-7 family miRNAs, which target the synaptosome associated protein 23 (*SNAP23*) gene, and loss of SNAP23 expression interferes with the release of exosomes carrying antimicrobial-peptides [7].

As with other intracellular pathogens, *Cryptosporidium* infection influences apoptosis. Microarray analysis of 51 apoptosis-associated genes indicated biphasic regulation by *Cryptosporidium*, with an anti-apoptotic state at 6 and 12 h post-infection (hpi) and a moderately pro-apoptotic state at 24, 48 and 72 hpi [14]. Inhibition of apoptosis in infected cells increases parasite survival and continuing apoptosis in uninfected bystander cells act to decrease the host immune response and may contribute to evasion of host defenses [15]. Previous studies have reported that *Cryptosporidium* inhibited of host-cell apoptosis by activating NF-κB [16, 17]. Little is known about the regulation of host-cell apoptosis by miRNAs following *Cryptosporidium* infection. A previous study found that downregulation of miR-513 was followed by the upregulation of B7-H1 expression and decreased apoptosis [11].

Our previous study of the miRNAs expressed in HCT-8 cells infected with *C. parvum* found that miR-942-5p, miR-181d, miR-3976, miR-18b-3p, miR-34b-5p and miR-3591-3p may regulate apoptosis in the early phase of infection [18]. This study investigated the upregulation of miR-942-5p in cultured HCT-8 human ileocecal adenocarcinoma cells following activation of the TLR2/TLR4-NF-κB signaling pathway by *C. parvum*.

## Methods

### *Cryptosporidium* oocysts and HCT-8 cells

*Cryptosporidium parvum* subtype IIdA19G1 oocysts were maintained in infected neonatal calves and stored in 2.5% K_2_Cr_2_O_7_ solution at 4 °C after purification. As previously described, oocysts were excysted in 0.25% trypsin and 0.75% sodium taurocholate for 1 h with mixing every 5 min, followed by incubation at room temperature for 30 min [19, 20]. HCT-8 human ileocecal adenocarcinoma cells (American Type Culture Collection, Manassas, VA) were maintained in Dulbecco’s modified Eagle’s medium (DMEM) supplemented with 10% fetal bovine serum, 4 mmol/l l-glutamine, 100 U/ml penicillin, and 100 U/ml streptomycin at 37 °C in a humidified 5% CO_2_ incubator [18]. Cell monolayers in 24-well cell culture dishes were inoculated with 2.5 × 10^6^ purified sporozoites per well in DMEM. The sporozoite:host-cell ratio was 10:1.

### Real-time quantitative PCR (qPCR)

HCT-8 cells were washed three times with phosphate buffered saline (PBS) before adding 1 ml TRIzol reagent (Invitrogen, Waltham, MA, USA) to each well. Total RNA was isolated following the kit manufacturer’s instructions subsequent to treatment with Recombinant DNase I (Takara, Kyoto, Japan). RNA was reverse transcribed to cDNA with SuperScript IV Reverse Transcriptase (Invitrogen) by oligo (dT) and random primers. The cDNA was amplified using the TB Green *Premix Ex Taq* II (Takara, Kyoto, Japan) and the gene-specific primers shown in Table 1. GAPDH or β-actin genes were internal references for toll-like receptors (TLRs), the U6 gene was the internal reference for miR-942-5p. miR-942-5p was reverse transcribed to cDNA using the stem-loop primer (5′-GTC GTA TCC AGT GCA GGG TCC GAG GTA TTC GCA CTG GAT ACG ACC ACA TGG C-3′) and the primer (5′-CGC TTC ACG AAT TTG CGT GTC AT-3′) for U6. PCR included one 30 s cycle at 95 °C, 40 cycles of 5 s at 95 °C, 10 s at 55 °C, and 15 s at 72°C, and a final 15 s cycle at 95 °C, 1 min at 60 °C, and infinite at 25 °C. The Cq values were analyzed using the comparative Cq (ΔΔCq) method and the amount of target was obtained by normalizing to internal reference and comparing with the control group.

**Table 1:** Primer sequences used in qPCR

Target mRNA	Primers	
	Forward	Reverse
TLR1	GGTGTTGGCTGTGACTGTGA	TGGAGTTCTTCTAAGGGTATG
TLR2	GATGCCTACTGGGTGGAGA	AGACGGAAATGGGAGAAGT
TLR3	CCAAGCCTTCAACGACTG	TTGCGTGTTTCCAGAGCC
TLR4	CCGCTTCCTGGTCTTATCAT	TCTGCTGCAACTCATTTCAT
TLR5	CAACCTTACAGCGAACC	AAACATCCCAACAGAGC
TLR6	CAGTTAATACTTTAGGGTGCT	CGTTTCTATGTGGTTGAGGG
TLR7	CCTTTCCCAGAGCATACAGC	GGACAGAACTCCCACAGAGC
TLR8	CAGAGCATCAACCAAAGCAA	GCTGCCGTAGCCTCAAATAC
TLR9	GTGCAGCCGGAGATGTTT	CGTGAATGAGTGCTCGTGGTAG
TLR10	GCCCACCACAATCTCTTCCA	GCCCACATTTACGCCTATCCTT
GAPDH	AGAAGGCTGGGGCTCATTTG	AGGGGCCATCCACAGTCTTC
U6	GCTTCGGCAGCACATATACAAAAT	CGCTTCACGAATTTGCGTGTCAT
β-actin	AGCGAGCATCCCCCAAAGTT	GGGCACGAAGGCTCATCATT

### Western blotting

HCT-8 cells were grown to 80% confluence in 6-well culture plates and exposed to *C. parvum* sporozoites. The cells were lysed with a total protein extraction kit (Solarbio Life Sciences, Beijing, China), and the protein concentrations were determined with a Pierce Bicinchoninic Acid (BCA) Assay Kit (Thermo Fisher Scientific, Waltham, MA, USA) following the manufacturer’s instructions. The proteins in 30 µg samples of lysate were separated by sodium dodecyl sulfate polyacrylamide gel electrophoresis (SDS-PAGE) and blotted onto nitrocellulose membranes. Membranes were incubated with TLR4, NF-κB, IκBα, and β-actin primary monoclonal antibodies (Abcam, Cambridge, UK), and then with 0.2 µg/ml horseradish peroxidase (HRP)-conjugated secondary antibodies. The blots were read by an electrochemiluminescence (ECL) substrate (Thermo Fisher Scientific).

### Short-interfering (si)RNA

SiRNAs targeting TLR-2 and TLR4 mRNAs were designed by the Sangon Biotech (Shanghai, China). HCT-8 cells were grown to 60–70% confluency in 12-well cell culture plates and transfected with siRNAs using Lipofectamine 3000 (Thermo Fisher Scientific). The extent of inhibition was determined by qPCR assays of TLR2 and TLR4 expression at 48 h post-transfection. The siRNAs that caused the greatest inhibition of TLR2, TLR4 expression were TLR2, GGA AGA UAA UGA ACA CCA ATT (sense) and UUG GUG UUC AUU AUC UUC CTT (antisense); TLR4, CCA GGU GCA UUU AAA GAA ATT (sense) and UUG GUG UUC AUU AUC UUC CTT (antisense). The siRNA oligonucleotides had no significant overlap with homologous gene sequences. Nonspecific siRNAs containing the same nucleotides in an irregular sequence were used as controls. The siRNAs were labeled with Cy3 using a silencer siRNA labeling kit (Thermo Fisher Scientific) for identification of transfected cells by confocal microscopy. HCT-8 cells were infected with *C. parvum* sporozoites 6 h after siRNA transfection. Total RNA was extracted at 0, 4, 8, 12, 24 and 48 hpi.

### Inhibitors

Pyrrolidine dithiocarbamate (PDTC) and SC-514 (MedChemExpress, Monmouth Junction, NJ, USA) were used to inhibit NF-κB activation [21, 22]. HCT-8 cells were pretreated with inhibitor for 2 h prior to *C. parvum* infection. PDTC and SC-514 were used at concentrations of 3.286 μg/ml and 22.43 μg/ml, which were not cytotoxic in either HCT-8 cells or *C. parvum* sporozoites.

### Data analysis

Data are represented as the mean ± standard deviation (SD) from three independent experiments. Each independent experiment was conducted by three replicates of qPCR and the mean value was used for data analysis. One-way ANOVA or t-test was carried out using the software of GraphPad Prism version 8.02 (https://www.graphpad.com/).

## Results

### *Cryptosporidium parvum* activation of TLR2 and TLR4 in HCT-8

**Fig. 1:**
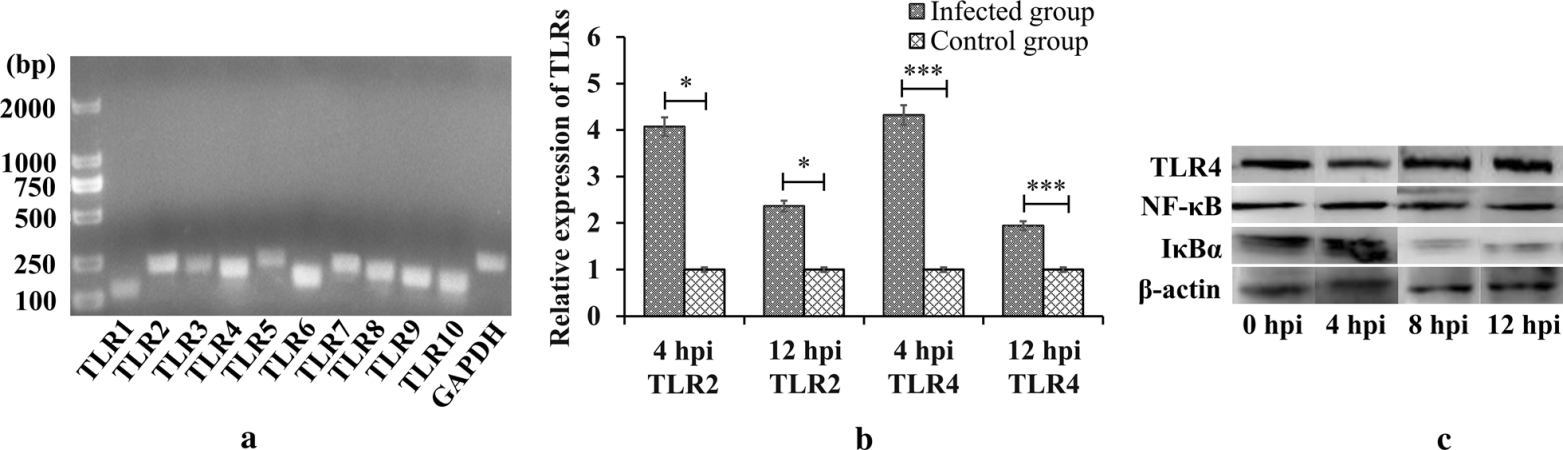
Promotion of TLR and NF-κB signaling molecules in HCT-8 cells by *C. parvum*. **a** Gel electrophoresis of qPCR products. **b** Expression of TLR2 and TLR4 at 4 and 12 hpi. Non-infected cells represent the control group (*P < 0.05 and ***P < 0.001 by t-test: test *versus* non-infected cells). **c** Western blots of TLR4, NF-κB, and IκBα protein at 4, 8 and 12 hpi; 0 hpi represents the control group

TLR1 to TLR10 expression was assayed by qPCR at 8 and 12 hpi. All were expressed in HCT-8 cells (Fig. 1a), but significant differences in infected and uninfected cells were observed only for TLR2 and TLR4. The difference was the greatest for TLR4 (TLR2 4 h: *t*_(4)_ = 4.961, *P* = 0.0077; TLR2 12 h: *t*_(4)_ = 4.052, *P* = 0.0155; TLR4 4 h: *t*_(4)_ = 22.31, *P* = 10^−9^; and TLR4 12 h: *t*_(4)_ = 12.18, *P* = 0.0003 by t*-*test: test *versus* non-infected cells) (Fig. 1b). Activation of the TLR/NF-κB signaling pathway was confirmed in western blots, which showed that expression of TLR4 and NF-κB increased and that of IκBα decreased at both 8 and 12 hpi (Fig. 1c).

### Upregulation of miR-942-5p by *Cryptosporidium parvum*

**Fig. 2:**
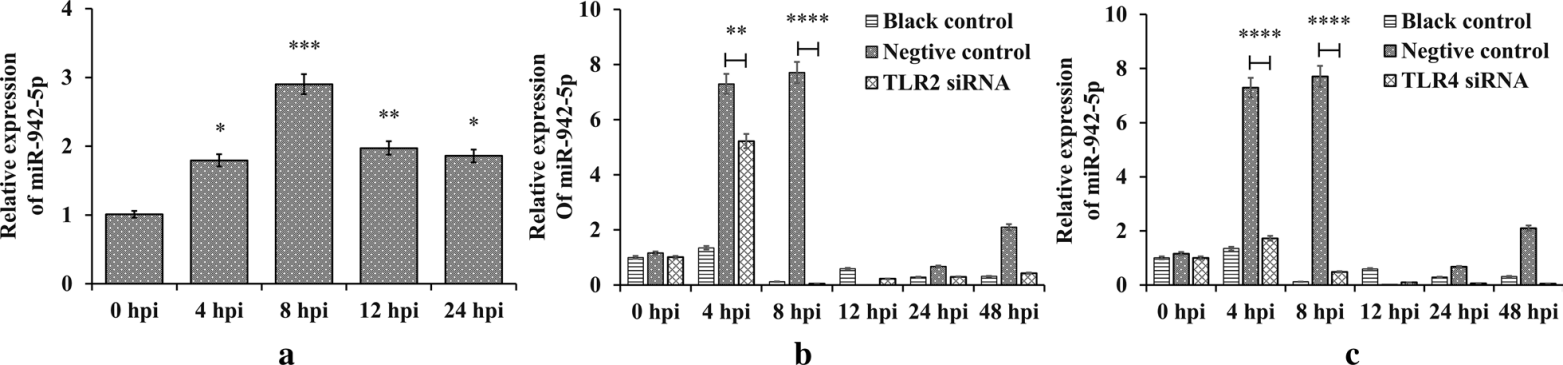
miR-942-5p expression in HCT-8 cells. **a** miR-942-5p expression in HCT-8 cells infected by *C. parvum* (0 hpi represents the control group). **b** miR-942-5p expression in *C. parvum*-infected HCT-8 cells after transformation with TLR2-specific siRNA. **c** miR-942-5p expression in *C. parvum*-infected HCT-8 cells after transformation with TLR4-specific siRNA. Black control represents the group of non-infected cells (*P < 0.05, **P < 0.01, ***P < 0.001 and ****P < 0.0001 by one-way ANOVA: test *versus* control group)

The qPCR results showed that miR-942-5p expression was significantly increased at 4, 8, 12 and 24 hpi, with the greatest difference compared with the control at 8 hpi (*F*_(4, 10)_ = 21.00, 4 h: *P* = 0.0121, 8 h: *P* = 10^−9^, 12 h: *P* = 0.0032, and 24 h: *P* = 0.0073 by one-way ANOVA: test *versus* control group) (Fig. 2a).

### Both TLR2 and TLR4 contribute to upregulation of miR-942-5p

Our previous study found that miR-942-5p was strongly upregulated in HCT-8 cells following *C. parvum* infection, compared with uninfected cells [18]. In this study, qPCR assays revealed that the transcription of the *miR-942* gene was significantly decreased after blocking either TLR2 or TLR4 (*F*_(2, 23)_ = 58.32, *P* = 10^−9^ by one-way ANOVA: test *versus* control group for Fig. 2b; *F*_(2, 27)_ = 89.08, *P* = 10^−9^ for Fig. 2c) (Fig. 2b, c). TLR4 appeared to have a stronger influence on miR-942-5p transcription than TLR2.

### NF-κB p65 is required for the transcription of miR-942-5p

**Fig. 3:**
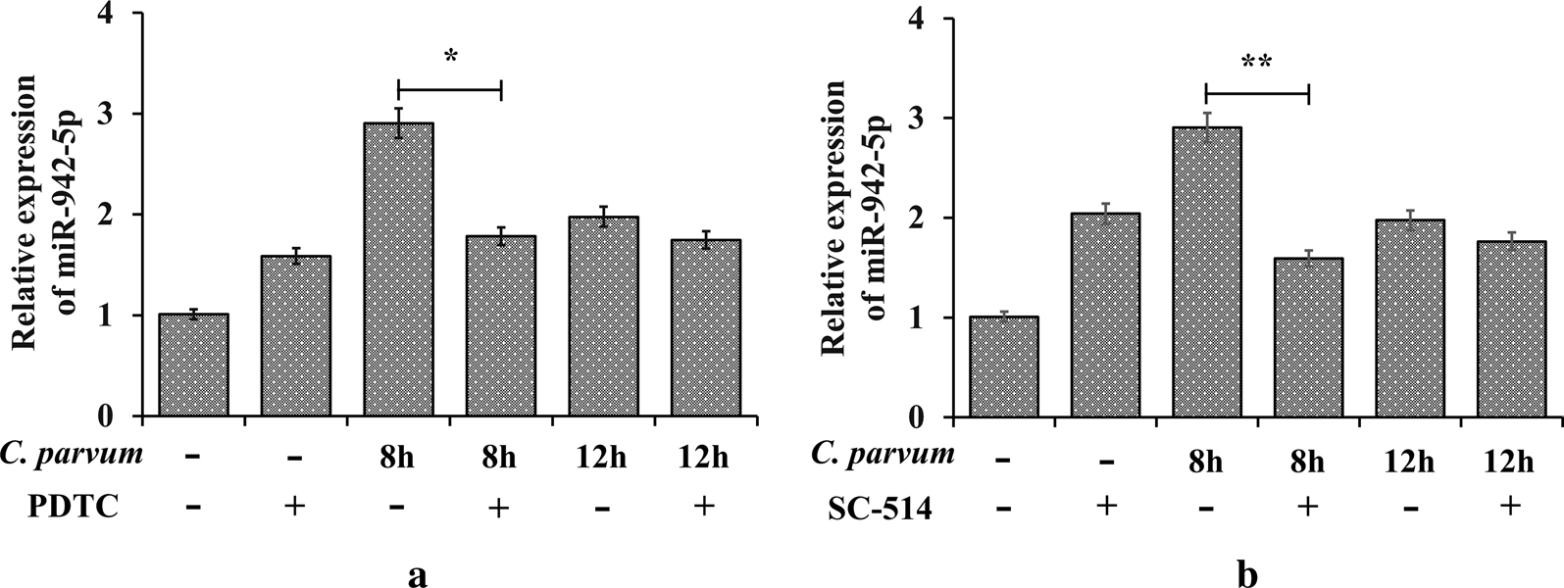
miR-942-5p expression in *C. parvum*-infected HCT-8 cells pretreated with NF-κB inhibitors. **a** PDTC inhibition of NF-κB. **b** p65-dependent inhibition of NF-κB p65 by SC-514 (*P < 0.05 and **P < 0.01 by t-test: test *versus* inhibitor-negative group)

Inhibition of NF-κB by PDTC resulted in downregulation of miR-942-5p expression at 8 and 12 hpi compared with controls (*t*_(4)_ = 4.200, *P* = 0.0137 by t-test: test *versus* inhibitor-negative group) (Fig. 3a). Inhibition of p65-associated transcriptional activation of the NF-κB pathway by SC-514, a nuclear factor kappa-B kinase-2 (IKK-2) inhibitor that prevents NF-κB-dependent gene expression, blocked the *C. parvum*-induced increase of miR-942-5p (*t*_(4)_ = 5.436, *P* = 0.0056 by t-test: test *versus* inhibitor-negative group) (Fig. 3b). Promoter binding of the NF-κB p65 subunit was thus required for the transcription of *miR-942* gene induced by *C. parvum* in HCT-8 cells.

## Discussion

Cultured HCT-8 cells expressed all known TLRs (TLR1-TLR10) and *C. parvum* infection induced the upregulation of TLR2 and TLR4, but not other TLRs, as was previously found in H69 human choanocyte cells [23]. Upregulation of TLR4 was stronger than that of TLR2 (Fig. 1b), but activation of either receptor recruited downstream components, with increased NF-κB expression and decreased expression of IκBα, an NF-κB inhibitor. Nuclear translocation of NF-κB activated transcription. TLR2- and TLR4-induced activation of NF-κB has previously been reported in H69 cells infected by *C. parvum* [23].

The upregulation of miR-942-5p after *C. parvum* infection was dependent on TLR2/TLR4-NF-κB signaling. TLR4 may have had a stronger effect than TLR2, especially at 4 hpi, but both TLR2 and TLR4 contributed to the upregulation of miR-942-5p expression (Fig. 2b, c). There are few data on the difference in the contributions of TLR2 and TLR4 during *C. parvum* infection, but TLR4-NF-κB signaling has been reported more frequently. TLR2 may be involved in *C. parvum*-induced stabilization of iNOS mRNA expression in biliary epithelial cells [13]. Post-transcriptional suppression of TLR4 expression by *let-7i* has been shown to contribute to immune responses to *C. parvum* infection in cultured human cholangiocytes, and mu-miR-92a-2-5p, which targets TLR2, relieves *Schistosoma japonicum*-induced liver fibrosis [6, 24].

A microarray analysis found that miR-942-5p was strongly upregulated during the early phase of *C. parvum* infection, and in this study qPCR confirmed that *C. parvum* infection was followed by significant upregulation of miR-942-5p at 4, 8, 12 and 24 hpi (Fig. 2a). Bioinformatics analysis indicated that miR-942-5p may be involved in the regulation of host-cell apoptosis. Previous studies have shown that miR-942 regulated cell apoptosis in response to microbial infection. For example, downregulation of miR-942 enhanced the apoptosis of HLCZ01 cells in response to hepatitis C virus infection [25]. Targeting of the *IFI27* gene by miR-942-5p has been shown to inhibit apoptosis role in HCT-8 cells during the early phase of *C. parvum* infection (our unpublished data).

## Conclusions

HCT-8 cells expressed all known TLRs, and TLR2 and TLR4 were upregulated following *C. parvum* infection with activation of downstream signaling. miR-942-5p was significantly upregulated after *C. parvum* infection, especially at 8 hpi, in response to a p65-dependent TLR2/TLR4-NF-κB signaling. TLR4 appeared to play a dominant role.

## Data Availability

Data are available from the authors upon reasonable request.

## Abbreviations

HCT-8 cells: HCT-8 human ileocecal adenocarcinoma cells
qPCR: real-time quantitative polymerase chain reaction
siRNA: short-interfering RNA
NF-κB: nuclear factor kappa-light-chain-enhancer of activated B cells
IκBα: nuclear factor of kappa light polypeptide gene enhancer in B cells inhibitor, alpha
PBS: phosphate buffered saline
TLRs: toll like receptors
SDS-PAGE: sodium dodecyl sulfate polyacrylamide gel electrophoresis
PDTC: pyrrolidine dithiocarbamate
